# Assessing Hand Sanitizer Usage in Japanese Elderly Day Care Centers: An Observational and Interventional Study

**DOI:** 10.7759/cureus.46834

**Published:** 2023-10-11

**Authors:** Masaki Takebayashi, Yudai Kaneda, Mira Namba, Akimi Yamashiro, Kurenai Takebayashi

**Affiliations:** 1 Sociology, Aomori University, Aomori, JPN; 2 Graduate School of Health Sciences, Aomori University of Health and Welfare, Aomori, JPN; 3 Medicine, Hokkaido University, Sapporo, JPN; 4 Medicine, Keio University, Tokyo, JPN; 5 Nutrition Science, Shokei Gakuin University, Natori, JPN; 6 Nutrition Science, Sendai Shirayuri Women’s College, Sendai, JPN; 7 Public Health, Olympus Co., Tokyo, JPN

**Keywords:** japan, covid-19, who guidelines on hand hygiene in health care, hand sanitizer, nudge

## Abstract

Purpose

Hand hygiene is a vital preventive measure against the novel coronavirus disease 2019 (COVID-19) infection. Though older people are at high risk of infection of COVID-19, there is still a lack of verification of hand sanitization and effective interventions to promote its implementation for older people. This study aimed to validate what kind of intervention can effectively promote hand hygiene among elderly individuals in Japanese day care centers, based on the hypothesis that a particular nudge will significantly promote hand hygiene.

Methods

We designed two types of interventions that have aspects of the nudge concept based on WHO Guidelines on Hand Hygiene in Health Care. The study was conducted with one-week observation and intervention periods excluding weekends, starting on February 11, 2023. Three groups were defined as follows: control (installation of hand sanitizer dispenser with a basic sign), reminder (additional visual cues using yellow curing tape), and institutional safety (sign appealing to protecting institutional safety by using hand sanitizer). Daily hand sanitizer usage was measured, and statistical analysis was performed using a one-way ANOVA for the number of uses before and after intervention.

Results

During the observation period, the average number of hand sanitizer pushes per person remained at 0.39 across all facilities. During the intervention period, control, reminder, and institutional safety groups showed increases of 1.13-fold, 1.31-fold, and 1.16-fold, respectively, revealing no significant difference.

Conclusions

Though these nudges were implemented according to WHO guidelines and the previous study, the older users of day care centers seemed not to react to these nudges significantly. The results underscore the need for tailored interventions to improve hand hygiene among the elderly in day care settings, contributing to the overall goal of preventing infectious diseases.

## Introduction

Since the first confirmed case in December 2019, the novel coronavirus disease 2019 (COVID-19) has spread worldwide, resulting in more than 68 million fatalities as of March 2023 [1]. The World Health Organization (WHO) has reported that effective strategies to prevent the spread of infection include staying at home, reducing contact with others, practicing social distancing, frequent and proper handwashing or hand sanitizing, and wearing masks [2]. In particular, the importance of hand sanitization is significant, as improving hand hygiene can reduce pathogen transmission in health care settings by 50% [3].

In Japan, the Ministry of Health, Labor and Welfare (MHLW) recommends hand sanitization and hand sanitizing stations be installed at the entrances of public institutions and stores [4]. Generally, the awareness of Japanese people regarding hand sanitization is high, and in a survey conducted by the MHLW in May 2020 during the initial COVID-19 outbreak, 85.0-95.0% of respondents reported that they were implementing infection control measures, such as hand sanitization [5]. However, the usage of hand sanitization among even the staff members of public health centers was much smaller than this percentage [6]. In general, Japan is categorized as a "nudge-cautious country," suggesting a potential resistance to the acceptance of nudges [7]. Indeed, although not conclusively attributed to cultural differences compared to other countries, a study in Japan revealed a correlation between mutual tendencies and hand hygiene self-regulation, emphasizing the significant gap between awareness of hand sanitization and actual behavior [8].

Moreover, age has been reported as the most significant factor in decreasing the survival rate of COVID-19, with this trend being particularly pronounced in individuals aged 65 years or older [9,10]. A survey conducted in the United States found that the older the individuals, the more likely they were to perceive COVID-19 as a significant crisis [11]. However, despite these perceptions and risks, it has been reported that elderly individuals, especially unmarried men, tend to neglect compliance with preventive measures against COVID-19 [12]. Another study found that older adults in Japan also had a lower tendency to use hand sanitizer compared to younger adults [13]. Thus, with Japan’s second-highest aging rate globally and no cost of individuals’ COVID-19 medical expenses until its classification change under the Infectious Disease Control Act on May 8, 2023, there was heightened concern not only that a mass infection among the high-risk elderly could overwhelm medical institutions, risking a healthcare system collapse [14], but also that self-restraint measures might lead to excess mortality among these elderly individuals [15]. Furthermore, it has been reported that the government's strategy regarding COVID-19 countermeasures for older people is also insufficient and remained unchanged, underlining the need to implement measures to promote their compliance [16,17].

In this context, the significance of preventive measures, especially hand sanitization, became evident in settings like day care centers frequented by the elderly, some of whom have declining cognitive functions, and in this context, the concept of nudge derived from behavioral economics has the potential to change the situation. Nudge is commonly known as a way to guide people's behavior in a socially desirable direction while maintaining their freedom of choice, and it has been reported to be effective in promoting positive behavior in health care and health fields [18,19]. Therefore, it is expected a method that can improve the importance of hand hygiene in COVID-19 infection prevention interventions without relying on compulsory measures or monetary incentives. Indeed, the effects of interventions using nudges on people’s awareness and behavior to prevent the spread of COVID-19 have been reported in several studies [6,20-22].

Though older people are at high risk of infection of COVID-19, there is still a lack of verification of hand sanitization and effective interventions to promote its implementation for older people. Therefore, the primary research question addressed in this study is what kind of intervention can effectively promote hand hygiene among elderly individuals in Japanese day care centers, based on the hypothesis that a particular nudge will significantly promote hand hygiene.

## Materials and methods

Settings and participants

This survey was conducted as a controlled experiment with three groups. After requesting research cooperation from a day care center for older people located in Towada city, Aomori prefecture, Japan, three day care centers for older people applied for participation. These centers were randomly assigned to the control group, reminder group, and institutional safety group using a randomization table.

Aomori prefecture, the study site, is located in the northern part of Honshu and has the second-highest rate of depopulation in Japan. In 2021, the percentage of its population aged 65 years and over was 34.4% (national average: 46.8%), and the projected increase in the aging rate up to 2045 is estimated to be the fastest in the country [23,24]. The average life expectancy for men and women in Aomori prefecture was 78.67 and 85.93 years, respectively (national averages were 80.77 and 87.01 years, respectively), both of which are the lowest in Japan [25]. Furthermore, the number of physicians per 100,000 population is 191.9, significantly lower than the national average of 234.7, indicating limited medical resources in the prefecture. Moreover, Towada city was one of the earliest regions in Aomori prefecture to experience the outbreak of COVID-19, and its medical institutions have been repeatedly challenged by cluster infections. Consequently, Towada city was identified as a region requiring effective measures for COVID-19 prevention.

WHO guidelines on hand hygiene in health care

WHO guidelines on hand hygiene in health care are designed to promote hand hygiene, and identify the following five important strategies to promote hand hygiene behavior: "system change," "education," "observation and feedback," "reminders," and "institutional safety" [26]. As resources are limited in many elderly care facilities, it is assumed that all interventions cannot be implemented. Since it takes time to change the present system and many Japanese people already understand the importance of hand sanitization, it is presumed that "system change" and "education" may not have an immediate effect [5]. The remaining three interventions possess characteristics of nudges, and based on the recommendations of this guideline, we designed the following nudges for application in the research.

Intervention

An automatic spray-type hand sanitizer dispenser was installed at the entrance of each center. Based on a previous study [27], observation and intervention periods were set for one week each, starting on February 11, 2023, excluding weekends. As the centers had predetermined days for facility use, the comparison was made excluding holidays. No notices were posted during the first week, and the following intervention was implemented during the second week.

Control Group (No Nudges)

A single automatic hand sanitizer dispenser was installed along with a sign reading "Please use hand sanitizer."

Reminder Group

Given the difficulty of physical system changes in a center, a salient nudge was designed to improve visual access. Specifically, an arrow was drawn with yellow curing tape on the floor to make the location of the dispenser visible, along with a sign reading "Please use hand sanitizer." This applies to easy nudges [28].

Institutional Safety Group

A sign reading "Please use hand sanitizer to protect our center from a group infection" was posted to appeal to the consciousness that "using hand sanitizer protects the safety of our center” by showing the losses associated with not using hand sanitizer. This applies to social nudges [28].

The intervention for the reminder group was consistent with our previous research [6], and for the institutional safety group, the principles of "institutional safety" in the WHO guidelines were modified to fit the context of day care centers [26]. Note that all groups included an "observation and feedback" component because the users were informed that they were observing consumption and fed back the results. Specifically, we notified the users of the day care center that we were observing the amount of disinfectant used. After the completion of the research, we displayed the usage amounts for a two-week period as feedback.

Methodology and outcome measures

Daily, the number of users at the facility was recorded, and after closing, the quantity of hand sanitizer used was measured. The usage per person was then computed for each facility. As the automatic dispenser sprays 3 mL per use, the number of uses was calculated based on the consumption amount. Additionally, based on prior research and assuming an alpha error of 5%, a power of 80%, and an effect size of 40%, the sample size was determined to be 66.

Statistical analysis

The number of times the hand sanitizer was pushed per person was analyzed as the primary outcome. First, the assumption of normality in each group before the intervention was confirmed using the Shapiro-Wilk test, followed by a one-way analysis of variance (ANOVA) to ensure significant differences. Given that the day service center has fixed users for each day of the week and considering its utilization in a preceding nudge intervention study, we determined that one-way ANOVA would be appropriate for this analysis [29]. Subsequently, the 95% confidence intervals for the number of pushes per person before and after the intervention were calculated, and the differences between groups were assessed for statistical significance in the same way. Stata version 15.0 (College Station, TX: StataCorp LLC) was used for all data analyses, and a significance level of p<0.05 was deemed statistically significant. If necessary, we decided to perform Bonferroni correction for multiple comparisons.

Ethical approval

The interventions were conducted as part of facility management. We obtained approval from the institutions' managers and informed the users about the observations. The collected data did not contain any personally identifiable information. Following the study, we shared the results with the three respective groups. This research plan was approved by the Sendai Shirayuri Women’s College Research Ethics Committee (approval number: 2022-89).

## Results

The total number of users and the volume of hand sanitizer used during the observation and intervention periods across the three facilities were 335 persons and 390 mL, and 330 persons and 470 mL, respectively. Table 1 shows the hand sanitizer usage in each group throughout the study period.

**Table 1:** Usage of hand sanitizer in each group.

Variables		Observation period						Intervention period						
		Day 1	Day 2	Day 3	Day 4	Day 5	Sum		Day 6	Day 7	Day 8	Day 9	Day 10	Sum
Control group	Number of users (people)	20	17	19	19	17	92		17	18	18	18	18	89
	Daily consumption (mL)	20	30	15	10	20	95		25	25	20	25	10	105
	Pushes per person	0.33	0.59	0.26	0.18	0.39	0.34		0.49	0.46	0.37	0.46	0.19	0.39
Reminder group	Number of users (people)	20	27	23	20	25	115		22	24	23	20	24	113
	Daily consumption (mL)	35	40	30	30	30	165		40	60	35	40	40	215
	Pushes per person	0.58	0.49	0.43	0.50	0.40	0.48		0.61	0.83	0.51	0.67	0.56	0.63
Institutional safety group	Number of users (people)	26	26	25	24	27	128		26	29	23	26	24	128
	Daily consumption (mL)	30	20	20	30	30	130		30	30	25	25	40	150
	Pushes per person	0.38	0.26	0.27	0.42	0.37	0.34		0.38	0.34	0.36	0.32	0.56	0.39

**Figure 1:**
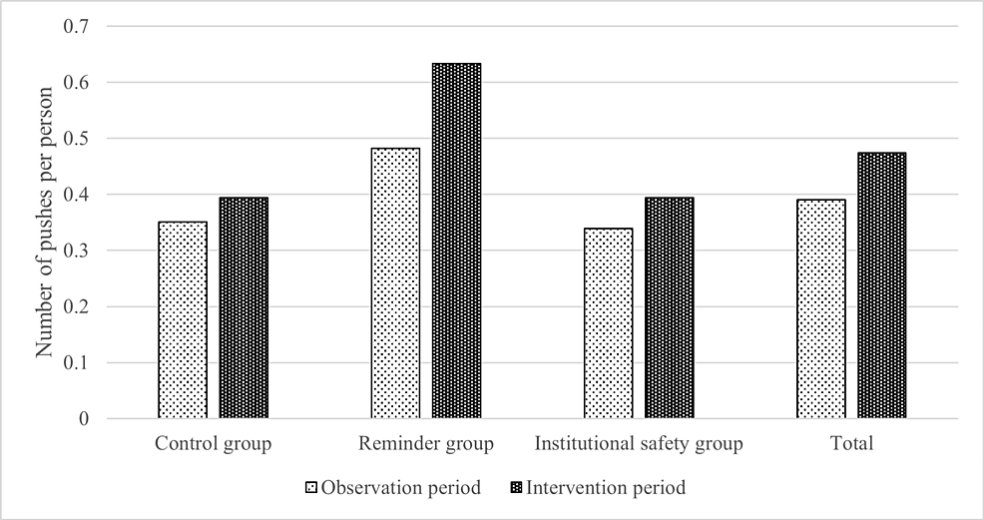
The number of pushes of hand sanitizer per person in each group. Control group vs reminder group, p=0.52. Reminder group vs institutional safety group, p=0.58. Control group vs institutional safety group, p=0.90.

In the control group, the total number of users was 92 during the observation period and 89 during the intervention period, with the total consumption of disinfectant amounting to 95 mL and 105 mL, respectively. In the reminder group, the total number of users was 115 during the observation period and 113 during the intervention period, with the total consumption of disinfectant amounting to 165 mL and 215 mL, respectively. In the institutional safety group, during both the observation and intervention periods, the total number of users was 128, with the total consumption of disinfectant amounting to 130 mL and 150 mL, respectively. Regarding the results of the observation period, the Shapiro-Wilk test was conducted for each group, yielding the following p-values: control group, p=0.835; reminder group, p=0.820; and institutional safety group, p=0.234. This indicates that all groups can be considered to follow a normal distribution. Furthermore, a one-way analysis of variance (ANOVA) was performed, resulting in a p-value of 0.103, confirming no significant differences among the three groups for the observation period. Overall, the average number of pushes per person was 0.39 and 0.47 in the observation and intervention periods, respectively. Figure 1 shows the number of pushes of hand sanitizer per person in each group.

When examined by group, during both the observation and intervention periods, the number of pushes was 0.34 and 0.39 (an approximate 1.13-fold increase after the intervention, 95% CI: 0.157-0.544 before, 0.239-0.550 after) in the control group, 0.48 and 0.63 (an approximate 1.31-fold increase {95% CI: 0.395-0.569 before, 0.477-0.791 after}) in the reminder group, and 0.34 and 0.39 (an approximate 1.16-fold increase {95% CI: 0.249-0.429 before, 0.277-0.510 after}) in the institutional safety group, respectively. Although such changes may partially implicate the use of hand sanitizers by the intervention, the p-values for the differences in changes were more than 0.05, confirming the lack of statistical significance as follows: control group vs reminder group (p=0.52), reminder group vs institutional safety group (p=0.58), and control group vs institutional safety group (p=0.90).

## Discussion

This study undertook controlled experiments in Japanese elderly day care centers aiming to uncover effective hand hygiene promotion nudges tailored for older individuals. While there was a 1.13-fold increase in hand sanitizer usage in the control group, the reminder and institutional safety groups saw a 1.31-fold and 1.16-fold increase, respectively. Such data points toward the potential efficacy of clear instruction displays in bolstering usage rates.

Intriguingly, despite the nudges in the reminder and institutional safety groups being designed based on WHO guidelines and prior research, their impact did not significantly differ from that of the control group [26-28]. This absence of significant results stands in contrast with other studies that have underscored the effectiveness of nudges in modifying health-related behaviors, including hand hygiene [30,31]. In fact, in a previous study we conducted targeting health care center users, the reminder nudge, in particular, resulted in a 42% points increase in consumption compared to the control group [6]. However, in the current study involving elderly participants from day care centers, the reminder nudge yielded only an 18% points increase, which is less than half the effect observed in health center users [6]. This reduced effect might be attributed to the elderly's diminished visual acuity or cognitive abilities, causing them not to notice indicators. Additionally, the limited efficacy might also be influenced by specific psychological or cultural nuances inherent to the elderly users of these centers [32]. Therefore, it is imperative to investigate alternative nudges or interventions tailored to the unique characteristics of Japan's older population as a preparation for the possible resurgence of future pandemics [33].

Of note, previous research has shown that older people may experience a stronger "present bias," potentially perceiving the act of sanitizing as burdensome and thus postponing it [34]. Also, older adults with cognitive decline, such as those with dementia, may have difficulties completing handwashing tasks [35]. Given the unique characteristics of the older population and the environment of the day care centers, the effects of a single nudge might be limited for older people, and a more personalized approach, including a combination of multiple nudges or utilizing assistive technologies, like the Cognitive Orthosis for Assisting Activities in Home (COACH) prompting system, might have been necessary to influence proper hand hygiene behavior [35,36].

The observed average number of hand sanitizer pushes per person, being 0.39 during the pre-intervention period, is an alarming revelation in the context of COVID-19 prevention among the older population in Japanese day care centers. Despite the high awareness regarding hand sanitization in the general population of Japan, this study uncovered a lack of compliance among older people. This observation resonates with previous reports that elderly individuals tend to neglect preventive measures against COVID-19 [37]. Indeed, the period in which this study was conducted, February 2023, corresponded with a time when reports indicated a decline in the number of COVID-19 infections, and discussions were also taking place regarding the reassessment of regulations related to the wearing of masks, which may have potentially affected their behavior [38,39]. Furthermore, due to the prolonged implementation of COVID-19 countermeasures, there is a possibility that the elderly have developed habituation to preventive behaviors [30]. However, older people were still exposed to a high risk of infection. The insufficient use of hand sanitizers reveals a gap between the magnitude of risk and actual behavior, emphasizing the urgent need for targeted interventions tailored to the behavior, perception, and needs of older people. Considering the elevated risk of COVID-19 complications among individuals aged 65 years and older, these findings underscore the importance of collaborative efforts to identify, understand, and overcome the obstacles to hand hygiene in such specialized environments [9,10]. Further research and policy formulation are required to address this gap and ensure that preventative measures effectively encompass all layers of the population, including the most vulnerable individuals.

This study has some limitations. One notable limitation of this study is the seasonal bias, beyond Japan's unique cultural and institutional context; given its timing during a period of low infection rates and pending relaxation of restrictions, combined with the cold climate, may have resulted in the cancellation of day care center visit and insufficient sample size [8,38-43]. However, while the interventions implemented in this study did not significantly alter hand sanitization practices among the elderly, previous research has shown that nudges can be over 10 times more cost-effective than education or incentives, and the observation in this study that hand sanitizer use increased with less than one dollar interventions is noteworthy given its potential real-world impact on reducing infections [44]. Additionally, the methods and findings provide a foundation for future research and interventions to enhance preventive measures against infectious diseases. Future studies may explore different types of nudges, conduct longer intervention periods, or focus on other target groups or settings where hand sanitization is crucial for their health management [40,42]. Furthermore, incorporating qualitative research methods, such as interviews or observations, could provide deeper context and insights into these areas. Thus, despite the limitations, this study's controlled experimental design in a real-world setting, especially considering the reported effectiveness of hand sanitizer use in infection prevention, adds value and provides a template for future research [3].

## Conclusions

This study was conducted in Japanese elderly day care centers according to WHO guidelines and the previous study but did not promote hand hygiene significantly. Despite not achieving the desired outcome, the findings provide insights into the complexities of nudging in older people, highlighting the need for personalized approaches. The study's limitations point to potential areas for future research, emphasizing the need for personalized approaches in promoting hand hygiene among the elderly, making it a valuable foundation for further exploration and innovation in public health strategies.
